# Omental Evisceration after Cesarean Section: Safety of Peritoneal Nonclosure Technique

**DOI:** 10.1155/2011/205437

**Published:** 2011-07-31

**Authors:** Sohini Bhattacharya, Sanjay Kumar Bhattacharyya, Hajekul Alam, Samir Chandra Ghosh Roy

**Affiliations:** Department of Gynaecology and Obstetrices, North Bengal Medical College, Shushrutnagar, Darjeeling 734012, India

## Abstract

A case of omental prolapse presented to us on the fifteenth postoperative day following an uneventful Cesarean section. A rare complication as such questions the safety of peritoneal nonclosure that has been adopted by obstetricians in recent times.

## 1. Introduction

One of the most significant changes that have been adopted in obstetric practices over the past few decades is undoubtedly the increasing frequency of Cesarean sections. Women are now four times more likely to have a Cesarean birth than 30 years ago. There is, however, wide variation in the surgical techniques used in Cesarean section and the quality of evidence to support the technique used. A rare complication often compels us to rethink and question the conventional surgical practices used.

We hereby present a case of omental prolapse, which questions the safety of non-closure of peritoneum at Cesarean sections.

## 2. Case Report

A 24-year primigravida lady underwent emergency lower segment Cesarean section for breech presentation in labour. Lower segment Caesarean section was done with low transverse abdominal incision under spinal anaesthesia. Uterus was sutured in two layers, and both the parietal and visceral layers of peritoneum were left open as per conventional practice. The rectus sheath was sutured in a continuous manner with polyglycolic acid suture. Skin was closed with nylon in interrupted mattress sutures. In early postoperative period she developed slight cough. Moreover, she complained of constipation during the first three postoperative days which resolved later. Otherwise the postoperative period was uneventful. Antibiotic prophylaxis was provided with intravenous Ceftriaxone twice daily. Analgesia was provided with intramuscular Diclofenac. Skin sutures were removed on the sixth postoperative day. She was discharged the next day.

**Figure 1:**
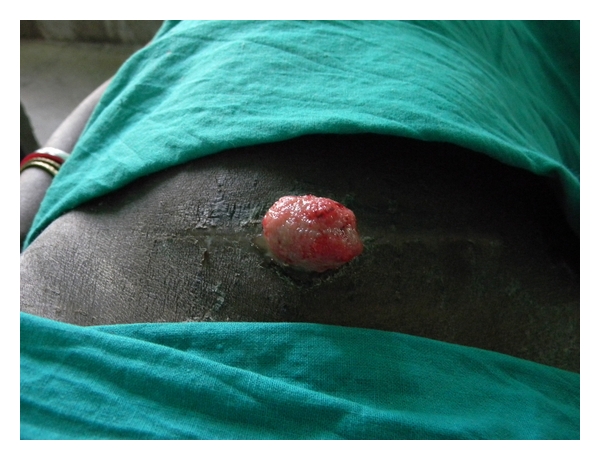
Omental prolapse through an apparently healed skin incision—on the fourteenth postoperative day.

**Figure 2:**
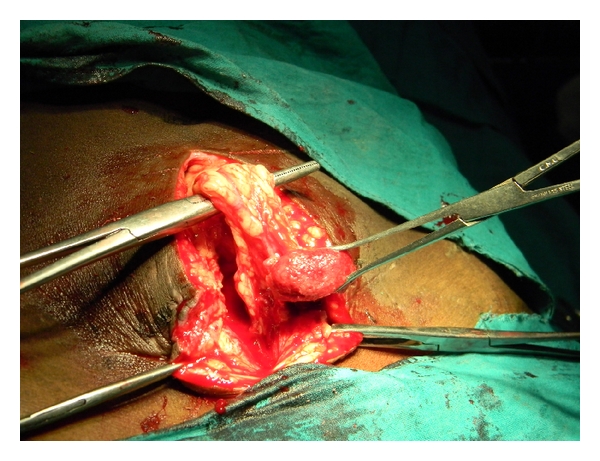
Evisceration of the omentum as revealed at laparotomy.

After one week she attended emergency and got admitted with the complaint of a fleshy growth arising from the Cesarean section wound. On examination her vitals were absolutely stable. Abdomen was soft, and peristaltic sounds were present. Local examination revealed a polypoid pink mass 5 cm × 5 cm protruding from midpoint of an otherwise healed Cesarean section wound (Figure 1). The mass was painless, felt soft in consistency and bled to touch. There was no associated discharge. It was attached to the deeper intra-abdominal structures. The skin was well healed on either side of the mass. Ultrasonography of abdomen revealed the mass as omental prolapse. Laparotomy was performed the next day. The portion of the omentum was separated and excised (Figure 2). The skin was closed after the parietal peritoneum, and rectus sheath was closed with polyglycolic acid sutures.

## 3. Discussion

Alternative surgical techniques to the traditional surgical approach to Cesarean birth have been reported in recent years. The Misgav-Ladach technique, developed in Israel, and the Stark technique adopted later involved the single layer uterine closure and non-closure of the visceral and parietal peritoneum.

The Cochrane Database Systematic Review showed that non-closure of the peritoneum reduced operative time. There was less postoperative pain and fever resulting in shorter duration of hospital stay [1]. But the other important outcomes were not adequately assessed, especially adhesions, effect on subsequent pregnancy, childbirth, or other surgery in later life.

Closure of peritoneum restores the anatomy by reapproximating the tissues, reduces infection by reestablishing an anatomical barrier, and reduces wound dehiscence and adhesions. Peritoneal healing is known to occur by migrating mesothelial cells with mesothelial matrix formation within the first five to eight days of surgery. It takes six weeks for the postpartum uterus to involute completely. Hence the postpartum enlarged uterus may act as a disruptive barrier to peritoneal healing if not reapproximated by sutures. Observational studies have suggested that there is more adhesion when peritoneal closure is not done in Cesarean sections. This leads to long-term morbidity like infertility and chronic pelvic pain [2].

There have been a few cases of evisceration of omentum following Cesarean section in the recent past. Omentum was sutured between the edges of fascia recti in one case [3]. Inadvertent injury to anterior rectus sheath during too much dissection of the same during surgery resulted in omental hernia in another [4]. Ruptured rectus sheath suture was the cause in a reported case [5].

Prolapse of omentum through a gap in the repaired rectus sheath at the end of the section might have occurred in early postoperative period due to coughing or straining at stool by the patient herself. This was small enough to escape notice of the intern at the hospital who removed the skin sutures.

Rapid growth of the omental mass occurred following discharge of the patient from the hospital to become noticeable.

All these cases reaffirm the technique of parietal peritoneal closure and drawing of rectus muscles, which are vertical breaks, so that these sutures close transverse incisions of the abdominal wall with cross sutures, which are very secure.
